# Immunosenescence, Aging, and Systemic Lupus Erythematous

**DOI:** 10.1155/2013/267078

**Published:** 2013-10-24

**Authors:** Gladis Montoya-Ortiz

**Affiliations:** Center for Autoimmune Diseases Research (CREA), School of Medicine and Health Sciences, Universidad del Rosario, Carrera 24 No. 63C-69, Bogota, Colombia

## Abstract

Senescence is a normal biological process that occurs in all organisms and involves a decline in cell functions. This process is caused by molecular regulatory machinery alterations, and it is closely related to telomere erosion in chromosomes. In the context of the immune system, this phenomenon is known as immunosenescence and refers to the immune function deregulation. Therefore, functions of several cells involved in the innate and adaptive immune responses are severely compromised with age progression (e.g., changes in lymphocyte subsets, decreased proliferative responses, chronic inflammatory states, etc.). These alterations make elderly individuals prone to not only infectious diseases but also to malignancy and autoimmunity. This review will explore the molecular aspects of processes related to cell aging, their importance in the context of the immune system, and their participation in elderly SLE patients.

## 1. Introduction

Aging can be defined as the progressive decay of tissue functions which eventually results in organ dysfunction and death. This decline may be the result of the loss of postmitotic cell function or the lack of replacement of such cells due to a decreased stem cell ability to maintain cell division and replication [1]. If the organism suffers damage and it is irreparable, the senescence or aging process will take place by limiting the cells' proliferative potential. Some control mechanisms include differential gene expression which may be detrimental [2]. However, there is a renewal mechanism that ensures damaged cell replacement. This singular mechanism corresponds to a set of proliferating precursor cells that provide a source of cell replacement within the tissues. The immune system provides an interesting case of replacement: cells that die by apoptosis are replaced by new ones, a process which is essential for immune system longevity and for adequate functionality. This review will describe main molecular mechanisms implicated in immunosenescence and their relationship with autoimmune disease, particularly related to systemic lupus erythematous (SLE).

## 2. Aging Molecular Mechanisms

**Figure 1:**
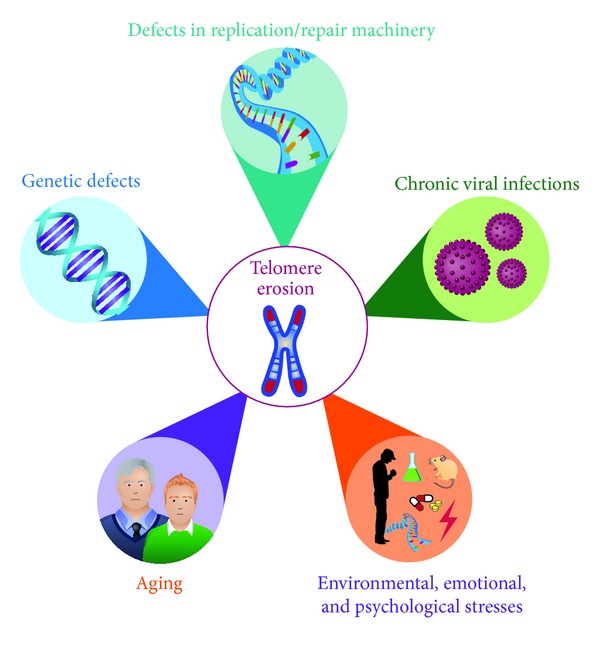
Factors related to telomere erosion. The mechanisms contributing to the loss of telomere length include genetic defects, chronic viral infections, defects in repair machinery, aging, and stress.

One of the most striking features of cell aging is its close relationship with telomere length [3]. There is an inverse relationship between telomere length and cellular aging; for example, very short telomeres force their cells to enter senescence. Human telomeres contain guanine-rich repetitive sequences (i.e., TTAGGG) which are gradually lost in each mitotic division. This occurs by the fact that the DNA polymerase is unable to replicate linear chromosomes in a process known as telomere erosion (Figure 1) [4–6]. This process functions as a mitotic clock for which the length of the telomeres represents the number of cell divisions sustained by the cells [7].

There is also a significant variability with respect to the speed and quality of aging between and within populations [8]. This heterogeneity results from interaction between genetic, environmental, and stochastic factors. In this regard, several epigenetic alterations have been associated with aging and diseases caused by aging (e.g., DNA methylation state, histone modification, miRNA, etc.) [9]. Several studies about DNA methylation have shown loss of methylcytosines with age, especially in CpG islands within Alu repetitive sequences and endogenous retroviral sequences. On top of this, a study of monozygotic twins showed that, for other young people, they retain similar methylation profiles while other twins who were between 50 and 60 years old had different methylation profiles and an H3 and H4 differential acetylation state [10]. 

Another mechanism related to epigenetic changes in aging involves chromatin remodeling. This includes H3K9, H3K27, and H4K20 trimethylation, decreased H3K9 acetylation, and increased H3S10 phosphorylation [11]. A decrease in H3K27 methylation together with an augmentation in H3S10 phosphorylation supports the idea of a change in the heterochromatin and euchromatin dynamics in aging cells. In addition, there are several chromatin remodeling-related proteins that suffer alterations during aging. These include the histone deacetylases (HDACs), the sirtuin 1 (SIRT1) protein, and the histone methyltransferases [12].

Finally, several studies in both murine and humans have shown that miRNAs may influence aging and longevity. Recently, multiple miRNAs related to aging have been described including lin-4, miR-1, miR-145, miR-140, miR-34a, and miR-449th, and some of them modulate cell senescence critical molecules such as class I HDAC, SIRT1, p21, p53, and pRb. Another important miRNA related to TCR signaling (miR181a) has serious implications in elderly people and autoimmunity (this topic will be discussed later). Recently, Liu et al. summarized the miRNAs involved in cell senescence [13].

## 3. Immunosenescence

One characteristic of elderly people is their inability to respond properly to vaccines and infections. This condition could be the result of their low immune system efficiency [14] and occurs because of thymic involution in which the thymus loses its ability to produce and replace naïve T cells on the periphery. As a result, thymic dysfunction produces a decrease in cell-mediated response to foreign antigens, self-tolerance, and naïve T-cell population. In turn, it could increase the autoproliferation of T cells and eventually the induction of premature T-cell maturation which would also conduce to tolerance reduction [15]. These alterations lead to not only modifications in lymphocyte subsets but also to functional changes in cell population subsets. For instance, longitudinal studies have demonstrated an association between immunosenescence and an increase in cytomegalovirus (CMV) anergic CD8 T cells [16].

One of the main characteristics of the immune system is the constant renewal of its cells. At the same time, this renewal is highly dependent on the efficiency of telomere maintenance. Immune system cells are derived from hematopoietic progenitor cells that come from myeloid and lymphoid lineages. These cells are constantly dividing and differentiating throughout their lifespan and that leads to changes in their telomere length. Note that a high rate of telomere loss in the first years of life has been observed, perhaps because of their high rate of mitotic division. However, this telomere loss is not a linear process over time since, in older people, it is possible to find significant telomere erosion. In fact, several studies have shown a decline in the length of telomeres with aging [17]. A lot has been written regarding the relationship between aging, thymic degeneration, and changes in the bone marrow cells [18–20]. However, we will focus only on immune senescence with an emphasis on circulating cell populations.

There are reports about age-related changes in peripheral blood cell populations: increase in monocytes, decreased lymphocytes, decrease in naïve cells, and increase in memory cells (Table 1) [21]. Curiously, memory T cells (CD4^+^-CD45RO^+^ and CD8^+^-CD45RO^+^) increase with age and are preferentially located in tissue, whereas there is a similar proportion of CD45RA^+^ and CD45RO^+^ subsets in peripheral blood [22]. Unlike somatic cells, lymphocytes have a robust capability to proliferate given their clonal expansion and present an overexpression in the telomerase. This process ensures no significant telomere shortening during each division. Interestingly, T cells possess several special features regarding their phenotype and their telomere length. T-cell memory cells have shorter telomeres than naïve T cells, and CD28^+^ T cells have longer telomeres than CD28^−^ T cells. 

Immune aging or immunosenescence not only affects adaptive response but also has implications in the innate response (Table 1) [23]. It has been found that older individuals who exhibit a breakdown of their innate immune barriers such as epithelial skin barriers, lungs, and gastrointestinal tract could be vulnerable to a pathogen attack. Among the cell types involved in innate response, there are neutrophils, macrophages, and natural killer (NK) cells, which also suffer functional alterations through aging (Table 1). Immunosenescence also affects the response to immunization. There are several reports indicating low response to infectious agents in elderly individuals [24, 25]. Latent proinflammatory status in old subjects is due to involution of thymus with subsequent alteration of function and balance of naïve, effector, and memory cells. This status combined with the presence of common and cumulative viral infections in the elderly (such as cytomegalovirus and Epstein-Barr virus) produces overall responses, loss of ability to control infectious diseases, and decreased response to vaccinations. The cytokine environmental balance (i.e., decline in the INF*γ* : IL-10 ratio) in challenge condition (i.e., influenza or other viral infections) could decline the CD8^+^ cytotoxic ability, thus conducing to high IL-10 response to virus challenge. Therefore, vaccines that arouse inflammatory cytokines would be expected to enhance protection in elderly subjects.

### 3.1. Adaptive Immune Response

#### 3.1.1. T Cells

T lymphocytes suffer alterations due to aging. Most of the observed changes are attributed to alterations during the initial activation step of the T-cell receptor (TCR). There is evidence of alteration in the downstream signaling of the TCR in the case of elderly people. This includes a decrease in intracellular free calcium, deficiencies in protein kinase C translocation, low Lck, ZAP70 activation, NFAT impairment, NF-*κ*B translocation, low ras-mitogen activated protein kinase (MAPK) pathways, and a decrease in proteasome activity, [26]. These alterations have been demonstrated in both naïve and memory T cells [27].

TCR has the function to discriminate between self-antigens and respond to foreign peptides. This is caused by its activation threshold level, and therefore, the loss of TCR sensitivity is closely related to aging. As mentioned before, there is an important microRNA—named miR-181a—implicated in this phenomenon which controls the expression of several phosphatases related to the negative regulation of proximal CD4 TCR signaling events. Indeed, in a murine model, miR181a overexpression lowers TCR activation threshold and restores TCR ability to respond to autoantigens [28]. Note that miR-181a expression declines throughout life and shows a significant loss after the age of 70 [29].

**Figure 2:**
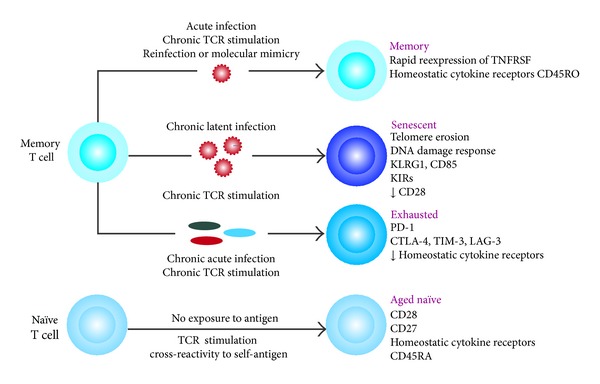
Changes in the T-cell pool and individual cells during aging. A Proportion of T subsets depends on individual infection antecedents and the environment. Memory T-cell subset can change to senescence by a chronic latent infection and chronic TCR stimulation. Meanwhile, exhausted T cells are produced by the same type of stimulation and the chronic acute infection. Aged naïve cells are generated from naïve cells stimulated by self-antigen exposure (adapted from [32]).

Another interesting point is that there are reports indicating changes in gene expression of surface molecules on T cells. Changes in surface molecules may have a negative impact on T-cell activation by increasing phosphatase expression such as DUSP family. Furthermore, a study evaluated the effect of aging on surface molecule gene expression and found that IL-6R, CD8, CD27, and CD28 are downregulated while ILT2 (CD85j), KLRG, KIR, CD44, CD96, Klrf1, and CD94 are upregulated [30, 31]. Some of these molecules (ILT, KLRG, and KIR) function as negative regulators of TCR activation and proliferation (at least in murine models) or as specific molecules of particular T cells such as cytomegalovirus (CMV) peptide-specific T cells (KIR and ILT2). They also appear to be related to T-cell exhaustion although the relationship between aging, senescent, and exhausted T-cell gene expressions seems to be different (Figure 2). An excellent review of this topic was done by Cavanagh et al. [32].

In addition to surface molecule gene expression alteration, there are other processes related to TCR signaling alteration and aging. For instance, it is well known that a cell-intrinsic environment (ROS species, DNA damage) and a cell-extrinsic environment (cytokines) both modulate TCR responses, and they are also altered in the elderly. Moreover, studies have shown that increased oxidative stress produces displacement of LAT from the cell membrane, thus inhibiting TCR signaling. Along with this, activated CD4 T cells from aging humans express increased levels of metallothoineins, which are an important redox system [34]. Additionally, there are alterations in molecules that participate in nuclear and cytoplasmic signaling pathways such as DNA repair kinases (ATM, ATR, and DNA-PKCs), which are activated not only by DNA double-strand breaks (DSB) but also by telomere attrition (as we will see later). Both of them are related to activation of DNA repair kinases (ATM, ATR and DNA-PKcs) [35].

Host environment and specific cytokine profiles have enormous implications in the T-cell signaling threshold. Some cytokines such as IL-7, IL-21, and IL15 have been studied in this regard. These cytokines signal through PI3K, STAT3, and STAT5 [36] and participate in ERK pathway activation. Note that IL-7 and IL-15 have profound implications in lymphopenia development in RA animal models. This has been shown through the fact that when animals are primed with IL-7 or IL-15, T-cell response to autoantigens is enabled. In addition, another cytokine implicated in signaling alteration during aging is the IL-6. This cytokine has implications in the JAK-STAT pathway through activation of SOCS3 transcription. Note that STAT molecules are highly phosphorylated in elderly humans, a phenomenon that also occurs through type I interferon T-cell activation [35].

**Figure 3:**
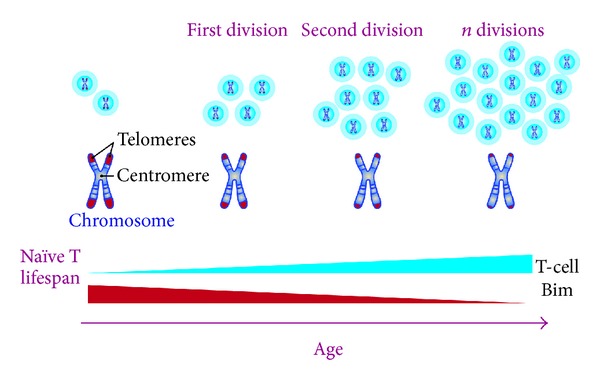
Schematic representation of T-cell divisions and their implication in telomere erosion and aging. Constantly dividing cells are accompanied by a decrease in their telomere length, which is related to aging phenotype: decreased Bim expression, increased naïve lifespan, and important functional changes.

As was discussed previously, telomere attrition is very frequent in elderly people and it is also known that T-cell replication is important for maintaining lymphocyte function. This suggests that T-cells employ the best mechanisms for telomere maintenance during clonal expansion. Indeed, there is evidence that telomerase activity is highly regulated during T lymphocyte development and differentiation [37]. The resting CD4^+^ and CD8^+^ and naïve T cells recorded no telomerase activity on the periphery. However, the telomerase is activated by lymphocyte stimulation. The level of telomerase activity decreases during successive stimulations of the lymphocyte (Figure 3) [38]. The rate of telomere shortening seems to be different among CD4^+^ and CD8^+^ cells, and it has been estimated to be 33 bp/year for CD4^+^ T cells and 26 bp/year for CD8^+^ T cells. One of the most outstanding features of aged CD4^+^ naïve T cells is their inability to produce significant levels of IL-2 after stimulation of their T-cell receptor (TCR). This inability subsequently leads to poor Th1/Th2 polarization. However, these cells may retain their ability to suffer Th17 differentiation [39, 40], which, in turn, could favor an inflammatory and autoimmune phenotype development. On the other hand, the number of CD4^+^CD25^+^FOXP3^+^ regulatory T cells (Treg) increases (2.4-fold), and they retain and gain functions during aging. Nonetheless, their ability to produce IL-10 is low. They may also contribute to Th17 bias (production of high levels of IL-17, IL-21, and IL-22) and show a decrease in antitumor responses too [40]. Meanwhile, aging CD8^+^ lymphocytes show a reduction in the diversity of the TCR repertoire, low antitumor response, and marked clonal expansion development but without the ability to replicate after stimulation [26]. Note that Th2 inflammatory cytokines favor antibody production by B cells, and this condition could explain autoantibodies in the aged population. 

One of the most important traits of immune aging is the loss of the CD28 surface marker. CD28 is one of the molecules expressed in T cells that provide costimulatory signals that are required for T-cell activation, T-cell proliferation, cytokine production, and T-cell survival promotion. Loss of CD28 expression is a phenotypic change associated with senescence in T lymphocytes, and it has been associated with functional alterations such as enhanced cytotoxicity, suppressive functions, and resistance of CD4^+^ T cells to apoptosis. Loss of CD28 expression is characterized by telomere shortening and reduced proliferative ability, both *ex vivo* and *in vitro* [41, 42]. At birth, virtually all T cells express CD28, but with age, the marker decreases about 40 to 50% for CD8^+^ T cells and 85 to 90% for CD4^+^. This reduction in the markers is attributed to repeated antigenic stimulation in peripheral blood [43]. However, when the CD28 is lost, cells suffer reprograming and, as a consequence, they express new receptors such as KIR, CD70, and perforin. Moreover, phenotypic CD28^−^ T-cell characteristics include interferon gamma (IFN-*γ*) production, potent cytotoxic capability, and CD158, CD158b, CD158J, DAP12, CD94, and CD244 receptor expression (similar to NK cell characteristics). These receptors give them the potential to interact with accessory cells such as mesenchymal cells, which include the fibroblasts of inflamed joints [44]. Furthermore, in elderly individuals with chronic viral infections and autoimmune diseases (e.g., multiple sclerosis (MS), rheumatoid arthritis (RA), and Wegener's disease), an increase in the frequency of CD28^−^ T cells has been detected [43]. It has been suggested that autoantigens can lead to clonal expansion of these cells. Thus, there are reports [45, 46] indicating how they can, for instance, show reactivity to myelin basic protein (MBP). The presence of CD4^+^ CD28^−^ T cells in both elderly individuals and patients with autoimmune diseases (ADs) has supported the concept that ADs are closely related to the cell aging process. In this regard, the loss of CD28 molecule could favor CTLA-4 interaction with their ligands (CD80 and CD86), which are implicated in autoimmune phenotype too. 

**Figure 4:**
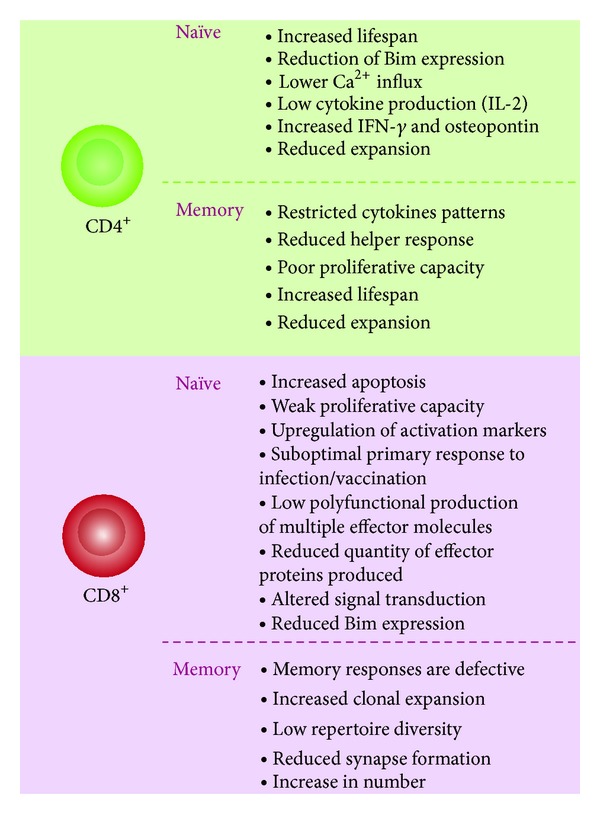
Aged-related functional changes in T-cell subsets. Alterations are produced in both memory and naïve subsets. These alterations depend on T-cell microenvironmental history, exposures to stressor agents, and stochastic events. There are differences in changes between CD4^+^ and CD8^+^ concerning aging, but in both cases, there is reduction of naïve subtype, increase in lifespan, and defective immune response.

The main differential and functional alterations of CD4^+^ and CD8^+^ T-cell subsets are summarized in Figure 4.

#### 3.1.2. B Cells

It is known that with aging, there is a decrease in not only the frequency and absolute number of pro-B cells but also in their ability to differentiate into pre-B (between 60 and 90%). Nevertheless, in healthy individuals, mature peripheral B-cell numbers do not change with aging; instead the relationship between naïve and memory cells is altered; that is, there is an increase in long-lived memory cells (homeostatic expansion of antigen-experienced or activated B cells) and a decrease in naïve cells [47]. This condition seems to depend on different factors other than genetic ones. A study comparing old individuals with healthy centenarian offspring could determine that centenarian offspring have more IgD^+^ CD27^−^ naïve B cells than older people. Nevertheless, the double negative memory cells (IgD^−^ CD27^−^ B cells) are only found in healthy elderly individuals, and there are no differences between groups [48]. Recently, studies have reported a novel peripheral B-cell subset in the elderly named aging-associated B-cell (ABC) subset (CD19^+^CD11b^+^CD11c^+^) [49]. *In vitro*, the ABC subset responds only to innate stimuli producing secretion of autoantibodies and cytokines, and this subset also has the ability to potentiate Th17 polarization, thus relating it to an autoimmune phenotype.

Another important fact is the presence of alterations in the repertoire of the B-cell receptor (BCR), which exhibits a decreased affinity and diversity to the antibody response with aging [33]. Indeed, elderly patients have impaired B-cell proliferation and activation, possibly as a result of defects in their threshold of activation [26]. Also, there is a loss of precision in distinguishing self- from non-self-antigens due to the oligoclonal expansion of the B lymphocyte subpopulation with a high proportion of antigen-experienced cells [50]. This subpopulation expresses CD5 on their surface, thus giving them the ability to produce low affinity antibodies independently of T cells. In the context of autoantibody generation, this is important for triggering an autoimmune response.

Moreover, the germinal centers (GC) from elderly people are small and have few cells producing IgM. In these individuals, levels of immunoglobulins, especially IgA and IgG, are increased [51]. Furthermore, it has been shown that IgG^+^/IgA^+^ B-cell subsets (both CD27^+^ and CD27^−^) express Ig mutated genes in their variable regions and high levels of CD80 and CD86 on their surface, thus exhibiting a similar B-cell memory phenotype [52]. According to the reports, this subtype of cells declines with aging [51]. Finally, aging B cells have been observed to produce antibodies with low avidity because of their somatic hypermutation deterioration which leads to a gradual decline in the humoral response. Nevertheless, repertory changes are not synchronous with aging, and decreased diversity has been related to poor health status [53].

An interesting study [54] done to evaluate naïve (CD19^+^CD27^−^) and memory (CD19^+^CD27^+^) switch B-cell subsets in elderly individuals showed a decrease in total B cells, and, although the quantity of naïve cells increased in percentage, they decreased or remained constant in number. Moreover, another striking result is that the B-cell memory (CD27^+^) increased in percentage but not significantly. In contrast, memory cells producing IgM subtype decreased in number but not in percentage. Finally, the memory switch cells decreased both in number and percentage with aging. An interesting point about memory B cells is that they have a hyporesponsive state to antigen-induced activation with less clonal expansion or less ability to differentiate into antibody secreting cells [55]. This condition may be caused by the decreasing number of antibody high affinity B cells that elderly people have. 

**Figure 5:**
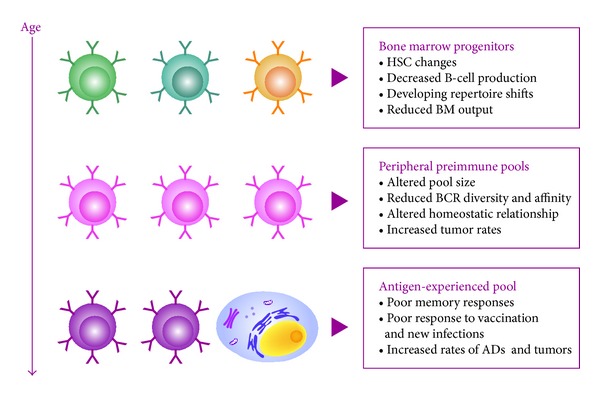
Age-related changes in the generation and function of B cells. There is a reduced output of B cells in the bone marrow, which induces accumulations in the periphery of antigen-experienced subsets with poor immune response and low diversity. HSC: hematopoietic stem cell; BCR: B-cell receptor; BM: bone marrow (adapted from [33]).

Taken together, these results indicate that there is an accumulation of antigen-experienced B subsets in aging individuals. In these cases, overall B-cell numbers are unchanged, but they vary in their functional abilities (Figure 5).

### 3.2. Innate Immune Response

#### 3.2.1. Dendritic Cells

Dendritic cells (DCs) are important because they function as a checkpoint between immunity and tolerance. DCs from aging individuals display a basal level of activation, increased secretion of proinflammatory cytokines such as TNF-*α* and IL-6 [56], and high levels of NF-*κ*B activation. However, they do not exhibit upregulation of CD86 and CD80 molecules on their surface, which suggests that they are partially activated. Another important characteristic is that they are more reactive to self-antigens compared with their young counterparts and display an impaired clearance of apoptotic cells and antigens [57]. This could produce a higher presentation of self-antigens and, consequently, an activation of autoreactive lymphocytes. An interesting point is that these partially activated DCs have a greater ability to stimulate T cells, thus indicating that their ability to induce tolerance to self-antigens is affected.

Some explanations have been given regarding partially activated DCs: (1) an increased age-associated level of proinflammatory mediators and (2) age-associated modifications in autoantigens, which increase their immunogenicity [58].

Note that the functions of myeloid DCs (mDCs) such as IL-12 production, chemotaxis, and their ability to activate naïve CD4 T cells via antigen presentation appear to be altered in elderly individuals [57]. This inability is due to decreased PI3K activation [59], which leads to activation of NF-*κ*B, as it was previously mentioned. This subtype of DCs also shows decreased capability in their antigen processing and increased expression of CD86.

Plasmacytoid DCs (pDCs) from elderly people, in turn, have reduced IFN I and IFN III production after stimulation via Toll-like receptor (TLR) [60]. Additionally, they have an impaired ability for antigen presentation to CD4 and CD8 T cells.

#### 3.2.2. Neutrophils

Neutrophils are the first immune cells that are recruited to the site of infection or to the tissue damage [61]. Besides, there is a debate about whether the numbers of neutrophils change with age, but a variety of studies suggest that there are no number changes. However, there are reports indicating several functional defects in neutrophils from the elderly [62, 63]. The main function decreased in neutrophils is the chemotaxis, followed by the phagocytic activity. Both of them affect the time needed for the neutrophils to reach the infection site and their ability to control the infections, respectively [62, 64, 65]. These two alterations are closely related to increased infections in elderly subjects. 

Low phagocytic activity has been associated with reduced surface expression of the Fc*γ* receptor CD16 [64]. Signaling function of other receptors involved in activation such as fMLp, TLR, retinoic-acid-inducible-gene-1-protein- (RIG-1-) like helicases (RLRs), nucleotide binding domain and leucine-rich-repeat-containing proteins (NLRs), and C3b has been reported to be significantly altered. This alteration is due to changes in signaling molecules but not in the number of their receptors [66, 67]. Some downstream signaling events include phosphoinositide-3 kinase (PI-3 K), MAP kinase, Calcium, protein kinase B, and SHP-1 and Jak-STAT pathways [68]. Interestingly, these alterations are produced by changes in membrane composition including lipid rafts distribution and their structure [69, 70]. 

#### 3.2.3. NK and NKT Cells

NK cells participate in the innate immune defense against intracellular pathogens and tumor cells, and they mediate MHC-independent cytotoxicity. There are several studies indicating a remodeling of these cells in elderly individuals. The percentage and absolute number of NK cells are increased in healthy aging, and they are characterized by the increment of CD57 expression and expansion of CD56dim NK cells (mature and highly differentiated cells) [71, 72]. Other important features of these cells from aging subjects are decreased proliferative response to cytokines, altered expression of some NK receptors such as natural cytotoxicity receptors (NCRs) [73], CD226 [74], and KLRG-1 [75], and increased expression of HLA-specific killer immunoglobulin-like receptor [73].

At functional level, cytotoxic and proliferation ability and cytokines/chemokines (such as INF*γ*, RANTES, MIP1a, and IL8) production of NK cells are reduced [76].

NKT cells are important in the clearance of bacterial and viral infections as well as in regulation of immune tolerance and autoimmunity [77]. NKT cells are characterized by expression of a TCR encoded by V*α*14/V*β*8.2 gene segments.

The effects of aging on NKT cell number and function have been little studied. In general, nowadays, it is accepted that the absolute number of NKTs within the lymphoid organ increases [78]. In addition, there are reports that show a decrease of proliferative ability and low number of CD1d-restricted NKT cells in the peripheral blood [79]. Studies of inhibition of NKT cell activation demonstrated age-associated decay of proliferative response and retarded type hypersensitivity responses [80]; in addition, results showed that NKT cells contribute to increments of IL-4 and IL-10 production and decreased IFN-*γ* in aging subjects [81]. 

#### 3.2.4. Monocyte/Macrophages

Other essential components of innate immune response are macrophages and monocytes. Monocytes respond to inflammation by their differentiation into macrophages and dendritic cells. Studies have demonstrated that CD56^+^ monocytes subpopulation (high producers of TNF-*α* via TLRs 2 and 4) is increased with age while their counterpart (CD56^−^) is decreased [82]. The increment of CD56^+^ monocytes is paradoxical with the alteration in macrophage TLR function. A study revealed a decrease in IL-6 and TNF-*α* via TLR1/2 in the elderly, and this fact was related to the decrease of TLR1 on monocyte surface [83]. These results are contradictory with another report which indicates substantial increase of the same cytokines [21]. Thus, this issue required further confirmation, which can be accomplished by the study of phenotype subpopulations (according to the expression levels of the receptors and proteins). In this regard, age-associated changes in TLRs expression on monocytes have been performed [84]. A particular study showed that old patients infected with West Nile virus have a persistent TLR3 expression on macrophages' surface while young patients have reduced expression of this receptor [85]. This feature may produce a higher inflammatory response with the subsequent increased morbidity of elderly subjects.

Furthermore, there are reports about age-related upregulation of CD80 molecule on monocytes after TLR activation [86] which is associated with production of a protective response to influenza vaccination.

Another important age-associated feature of both monocytes and macrophages is that several of their receptors become altered, thus producing cells dysfunction. This produces that clearance of free radical production and phagocytosis are reduced in monocyte/macrophages in the elderly [87]. Also, these alterations may lead to deregulation in clearing of apoptotic cells by macrophages, thus precipitating the exacerbation of inflammatory-aging condition.

## 4. Infection and Immunosenescence

To produce an adequate response to large numbers of pathogens throughout life, there are homeostatic mechanisms guaranteeing competent memory and a naïve cell pool for prolonging the survival of memory cells. However, under the conditions of advanced age, these mechanisms are seriously affected. As we have seen, during aging, many changes occur in the immune system, which means that immunosenescence becomes a factor contributing significantly to a higher risk and severity of infections. The most important diseases in the elderly are urinary tract infections, influenza and pneumonia, chronic viral infection reactivation (herpes virus and varicella-zoster virus), as well as bacterial (tuberculosis), fungal (candidiasis), or parasitic infections, and, more rarely, opportunistic infections such as *Clostridium* and *Staphylococcus* [88, 89]. Although the immune response to antigens may be preserved in elderly individuals, their ability to be immunized against new antigens is reduced. This may be the result of an increase in the proportion of memory cells and progressive decrease in naïve cells from the thymus [90].

While it is true that aging is associated with the emergence of infectious diseases, it is also true that these infectious events will promote aging. It is well known that viral infections (particularly the herpes virus family) are strong stressors which alter the lymphocyte phenotype and functionality, altered cytokine profile, resistance to apoptosis, and shortened telomeres [91]. These features are similar to those found in the elderly; thus it is possible that viral infections could represent an important extrinsic factor for aging by the repeated antigen stimulation characteristic of persistent latent infections [92]. Furthermore, it has been suggested that latent herpes virus infections are primarily responsible for *in vivo* generation of senescent CD8^+^ T cells, perhaps due to constant and prolonged virus-specific T-cell proliferation [93]. Additionally, the Epstein-Barr virus (EBV) latent infection has also been associated with telomere shortening in antigen-specific CD8^−^ T cells because EBV antigens cause a decrease in telomerase activity associated with T-cell proliferation [94]. In contrast (and related to telomere erosion and its relationship with CD28 molecule expression), the majority of T cells in a study done by Vescocini were CD28^+^ unlike what was found for CMV, which were mainly CD28^−^ [95]. During human immunodeficiency virus (HIV) infection, in turn, it has been reported that early presence of CD8^+^ CD28^−^ T cells is a predictive characteristic of rapid disease progression [96].

These data indicate that chronic infections during aging produce significant changes in the CD8^+^ cell subset. Additionally, this shows that expansion of CD28^−^ T cells is age dependent, and they have a positive correlation with proinflammatory cytokines. At the same time, these cytokines are heavily involved in the pathogenesis of immunological disorders which could favor the emergence of different pathologies including ADs. 

## 5. Autoimmune Disease

Currently, it is clear that changes occurring in the immune system during aging affect the onset of ADs. This is due to the fact that aging is related to increased reactivity to self-antigens and loss of tolerance. The overall tendency supports this hypothesis because elderly people experience general systemic inflammation and, at the same time, they aggravate degenerative diseases [97], which, in turn, increase the risk of developing ADs [98, 99]. Proinflammatory cytokines on general systemic inflammation (produced by viral infection) lead to a state called inflammaging, which corresponds to the loss of equilibrium between adequate inflammatory response and efficient anti-inflammatory control in the elderly condition. Later, in normal aging, this control fails to fully neutralize the inflammatory processes. 

In addition to this, it is important to remember (as we have seen previously) the epigenetic changes occurring in elderly people and how these may affect important genes involved in autoimmune disorder development [100]. In this regard, there are reports in which some genes associated with ADs are hypermethylated but others are hypomethylated. For instance, FoxP3, a hypermethylated gene, is a member of the forkhead transcription regulator family [101] and is associated with the development of multiple ADs. In contrast, the gene coding for the CD11a chain of lymphocyte function-associated antigen 1 (LFA-1)—a protein which is associated with certain ADs—is hypomethylated with age and thus overexpressed in aging cells [100]. Furthermore, there are other reports on elderly subjects indicating DNA hypomethylation states which could lead to an increase in the immunogenicity [58]

Another important aspect of aging that is closely related to autoimmunity in general and ADs in particular is the increase in inflammatory cytokines and chemokines such as TNF-*α*, C-reactive protein, IL-8, MCP1, and RANTES [102–104]. There is a substantial amount of evidence of age-associated alterations in the T-cell cytokine profile which could contribute to development of ADs. Studies have shown that there is a change from Th1 to Th2 molecules (mainly IL-4 and IL-6) in the cytokine profile as age advances [105]. IL-6 is a potent proinflammatory cytokine closely related to disability in patients with RA; therefore, IL-6 represents a therapeutic target for this disease [106]. In addition, there are reports of an imbalance between Th17 and Treg cells. A considerable number of IL-17-secreting naïve CD4^+^ T helper cells have been detected in the elderly in contrast to reduced IL-17-secreting memory CD4^+^ T helper cells [107]. 

Some ADs are very frequent in younger patients and are not limited to elderly people although the occurrence or presence of autoantibodies is greater at advanced age [108–111]. Autoantibody production such as rheumatoid factor, as well as antinuclear, antiphospholipid, and antithyroglobulin antibodies, is present during aging [109, 112]. Autoantibody production has been attributed to altered T- and B-cell functions [113], especially to the decrease in antibody affinity maturation. This evidence supports the idea that autoantibody levels may be closely related to the clinical characteristics of the elderly and to patients with ADs.

One of the important causes of dysfunctional immune responses is telomere abnormalities which may lead to autoimmunity. This observation is significant since numerous studies have shown an association between mean telomere length in peripheral blood mononuclear cells (PBMCs) and different diseases [91]. This evidence suggests an increase in CD8^+^ CD28^−^ T-cell proportions in several pathologies such as in the case of some ADs.

Moreover, there are reports of telomere length alteration in patients with ADs such as RA [114, 115], scleroderma (SSc) [116], systemic lupus erythematosus (SLE) [117], polyangiitis with granulomatosis [118], psoriasis, and atopic dermatitis [119], suggesting an excessive cell replication with their corresponding telomere erosion. These findings have been interpreted as evidence of T-cell accelerated proliferation in the autoimmune process.

At present, it is believed that there are differences among telomere abnormalities and various ADs. Some of these differences could be explained by the genetic background of the individuals studied. For example, a study performed in patients with SSc and their family members reported short telomeres [116]. The idea of a genetic predisposition to telomere shortening is also suggested in patients with RA, who exhibit telomere erosion in not only memory cells but also in naïve cells. Moreover, this evidence shows acceleration in telomere erosion occurring at the precursor cell level [120]. Another striking fact is that the genetic predisposition to short telomeres is strongly related to HLA-DR4 haplotype which is shared by RA and T1D in some individuals [121, 122].

### 5.1. Late-Onset Systemic Lupus Erythematous

**Figure 6:**
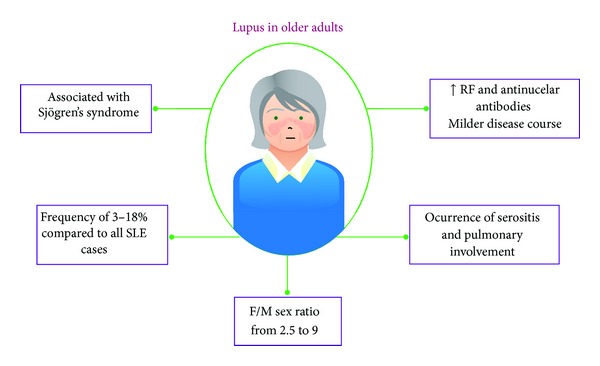
General characteristics of late-onset SLE. There are different manifestations of older SLE patients compared to young patients. RF: rheumatoid factor; F/M: female/male.

Although SLE is considered a disease of the reproductive stage of women, there is evidence that it occurs between 3 and 18% in individuals older than 50 years [123]. Despite that there are aged SLE patients, their clinical manifestations, response to treatment, prognosis, and course of the disease are different in these individuals (Figure 6). For example, clinical manifestations such as malar rash, renal disease, arthritis, and photosensitivity are less frequent in them, while serositis, cytopenias, and pulmonary involvement are more frequent [124–127]. In addition, it has been shown that female/male (F/M) sex ratio declines with age. Studies report F/M ratio from 2.5 to 9 in elderly individuals compared to from 9.1 to 14.4 in young people [126, 128].

A striking feature of these patients is the differential diagnosis due to the SLE overlapping with other diseases. Late-onset rheumatoid arthritis, endocarditis, tuberculosis, neoplasia, polymyalgia rheumatica, temporal arteritis, and Sjögren's Syndrome (SS) had been described is these patients [124, 127]. In the particular case of SS, elderly patients with SLE and without SS have low frequency of compromising renal disease, lymphadenopathy, and thrombocytopenia and high frequency of Raynaud's phenomenon [129].

Besides changes in clinical and serological profiles, serological profiles of aged SLE patients also exhibit alterations [124]. Compared to younger individuals, elderly patients with SLE have high frequency of rheumatoid factor (33% versus 20%) and antinuclear antibodies but low frequency of antiribonucleoprotein (anti-RNP, 10% versus 21%) and anti-Sm (9% versus 17%). There are reports that indicate contradictory results [130], but these differences can be explained by different reasons: ethnicity, sample size,  methodology, and so forth. 

An important fact is that the severity of the disease appears to decrease with age. It has been reported that late-onset SLE patients have milder disease course which is reflected in a small number of relapses per patient. Additionally, it was found that the prevalence of lupus nephritis and nephrotic syndrome also is lower in elderly patients.

Currently, it is not clear if there is a relationship between telomere loss and SLE. Some studies have shown an increased telomere erosion in SLE patients [100, 101, 131, 132], while others report normal telomere length when compared with healthy controls [102, 103]. Nevertheless, it is clear that there is a reduction in telomerase activity in naïve CD4^+^ T cells and an increased activity in B cells [101, 103]. In this regard, a recent study showed a differential expression of shelterin complex molecules in patients with lupus [104], but, unfortunately, it was not done cell specific. 

According to the report of [132], shorter telomeres are associated with Ro antibodies while longer ones with steroid therapy and increased body mass index. However this study also showed that short telomeres are not related to disease activity or immune cell turnover, but they could be good predictors of premature aging.

Related to this topic, previous studies have indicated that bone marrow mesenchymal stem cells (BMSCs) from SLE patients exhibit not only increased apoptosis and senescence but also impaired capacity of differentiation, immune modulation, proliferation, and secretion of cytokines. Apoptosis and senescence in BMSCs from SLE patients appear, to be due to increased favorable conditions for these processes. There were described increased levels of p16INK4A, Bax, caspase 8, Fas and tumor necrosis factor-a receptor 1, and the respective ligands of the two last. There is also a decreased expression of Bcl-2, CDK4, CDK6, and p-Rb [133, 134]. 

Programmed cell death (PCD) is an essential mechanism of homeostasis and development and it is very important as an immune response regulator. Hence appropriate clearance of apoptotic cells in the immune system is necessary for regulating inflammation and maintaining self-tolerance. Impaired clearance of apoptotic cells in patients with SLE is considered an important process in the etiology of lupus [135]. This phenomenon is exacerbated in older age, and its deficiencies may account for the development of autoimmunity by the loss of tolerance in lymphoid tissues. This process is mediated by phagocytes and, as it was mentioned before, in elderly subjects; phagocytic functions of macrophages are altered. This functional deregulation may generate danger signals, followed by concomitant exposure of autoantigens and the subsequent autoimmune reaction.

Another mechanism that is related to development of lupus is the molecular mimicry in which B and T cells are activated as a result of an infection. This mechanism makes cells able to recognize self-molecules that are similar to infectious agent molecules, thus originating an autoimmune response [136–139]. In SLE, this response is associated with high levels of anti-Sm autoantibodies due to similar molecular sequence of pathogenic molecules of CMV and EBV, which develop the initial immune response. Although anti-Sm autoantibodies are less frequent in the elderly than in younger individuals, it may be possible that EBV infection—exacerbated with age—can contribute not only to inflammaging development but also to induction, by molecular mimicry, of an immune response towards itself. Further studies in this topic could be interesting. 

Abnormalities in TCR signaling which have been documented in SLE patients are similar to those in RA patients. This may also be related to the elderly; for instance, TCR zeta chain expression is defective in these patients [105].

## 6. Concluding Remarks

Aging is a natural physiological process that could eventually conduce to increases in some pathological conditions. A lot of changes are detected in the immune system of elderly individuals which could contribute to the occurrence of complications such as infection, autoimmunity, and autoimmune disorders. In this regard, it is important to remember that elderly patients with SLE have different clinical and serological manifestations and poorer prognosis comparing with young patients (less insidious onset disease, more occurrences of severe manifestations, and higher frequency of comorbid conditions). Thus it is necessary to implement a proper immunological recognition of these patients in order to produce adequate therapeutic management which must be different because the treatment itself may cause long-term damage.

## Figures and Tables

**Table 1:** Age-associated changes in immune cell populations and functions.

Cell type	Characteristics	References
Innate immunity		
Neutrophils	↓ Phagocytic chemotaxis capability ↓ Superoxide anion production ↓ Ability to respond to soluble factors (GM-CSF) and bacteria (LPS and fMLP) ↓ Molecule recruitment into lipid raft, apoptosis, and signal transduction	[23, 70, 87, 140]
Dendritic cells	↓ Cell number, antigen presentation, TLR-mediated signaling, IFN I/III production, chemotaxis, and endocytosis ↓ Ability to stimulate lymphocytes in the ill elderly ↑ Function in the healthy elderly	[23, 102, 141]
Macrophages	↓ Phagocytic activity and chemotaxis ↑ Synthesis of proinflammatory cytokines (IL-6, IL-8, TNF-*α*, and IL-1*β*) ↓ Apoptosis, superoxide production, and signal transduction ↓ TLR expression and function ↑ PGE2 production ↓ MCH class II production	[23, 87, 142]
NK cells	↑ CD56dimCD57^+^ population ↓ Function of cytotoxicity ↓ Secretion of IFN-*γ* induced by Interleukin 2 (IL-2) ↓ HLA-DR, IFN-*α*, CD57, and CD95 ↓ Cell proliferation ↑ Production of IL-1, IL-4, IL-6, IL-8, and TNF-*α*	[72, 143, 144]

Adaptive immunity		
*Cellular response *		
Thymus	Involution from age of 9 months, thymic remnant after 50 years	[20, 145]
T Cells	Variable number (↓ proliferation to PHA, varying age, and health status)—HLA B8/DR3 associated with high proliferative responses ↑ Proportion of memory cells (CD45RO^+^), especially tissue CD8^+^ ↓ Proportion of naïve cells (CD45RA^+^) ↓ Proliferative capacity ↓ Synthesis of IL-2 receptor and IL-2 in memory cells ↓ CD28^+^ ↑ CD28^−^ T cells—mainly CD8^+^ CD28^−^ (characterized by oligoclonal expansion, shortening of telomeres, potentially decreased proliferation, resistance to apoptosis, and increased production of TNF-*α* and IL-6) ↓ CD4 T lymphocytes Change from Th1 response to Th2 response with ↓ cell-mediated responses directed against intracellular bacteria (Th1 function) and relative preservation of humoral response (Th2 function) ↓ Treg population (CD4^+^ CD25^+^) that plays a role in the manifestations of autoimmunity Impaired immunological synapse formation and signaling pathways (calcium response and phosphorylations) ↓ CD4/CD8 rate	[14, 145–147]
*Humoral response *		
B Cells	↓ Pre-B lymphocytes with peripheral B lymphocyte count unchanged ↑ CD5^+^ B cells (CD19^+^ CD5^+^ clones B) that produce low affinity antibodies without cooperation of T cell ↓ Naïve B cells Accumulation of memory B cells with ↓ diversity and affinity of antibodies Reaching primary humoral response (dependent T cell cooperation). Conserved secondary humoral response	[14, 33, 50, 53, 145, 148]
Immunoglobulins	↑ Serum levels of IgA and IgG (IgG1, IgG2, and IgG4). Monoclonal immunoglobulin production by CD19^+^ CD5^+^ clones. Secretion of non-organ-specific self-antibodies (rheumatoid factor, antinuclear antibodies, antiphospholipid antithyroglobulines, and parietal cells).	[51, 113]
Interleukins	↓ IL-2 production because of the following: ↓ cooperation of T cells with antibody producer B cells, ↑ production of IL-4, IL-6, IL-8, IL-10, and TNF-*α*, ↓ production of IL-1 and IFN-*γ*.	[14, 106, 145]
